# Incidence and Predictors of Bronchopulmonary Dysplasia Development and Severity Among Preterm Infants Born at 32 Weeks of Gestation or Less

**DOI:** 10.7759/cureus.59425

**Published:** 2024-04-30

**Authors:** Ahmed Abushahin, Sara G Hamad, Amal Sabouni, Sufwan Alomar, Anoop Sudarsanan, Hiba Kammouh, Prem Chandra

**Affiliations:** 1 Pediatric Pulmonology, Sidra Medicine, Doha, QAT; 2 Pediatric Pulmonology, Hamad Medical Corporation, Doha, QAT; 3 Neonatology, Hamad Medical Corporation, Doha, QAT; 4 Medical Research Center, Hamad Medical Corporation, Doha, QAT

**Keywords:** predictors, chronic lung disease of prematurity, prematurity, preterm, bronchopulmonary dysplasia (bpd)

## Abstract

Background

As the most common chronic lung disease (CLD) related to premature birth, bronchopulmonary dysplasia (BPD) is associated with long-term lung disease along with cardiovascular and neurodevelopmental disorders. However, data on the incidence and predictors of BPD in Qatar are lacking.

Objectives

In this study, we aimed to determine the incidence of BPD among infants born at ≤ 32 weeks gestational age (GA) at our neonatal unit, and identify risk factors for the development of BPD and moderate-severe BPD.

Methods

This was a retrospective observational cohort study conducted at a single site: a level-III neonatal intensive care unit (NICU) in Qatar. We included 1539 neonates born at ≤ 32 weeks of gestation with birth weights of ≤ 1500 grams who were admitted to the NICU between 2017 and 2020. Univariate and multivariate logistic regression analyses were performed to identify potential factors and predictors and their possible associations with the development of BPD and moderate-severe BPD. We also applied BPD classifications to determine the variability in the incidence of BPD in our cohort according to various definitions (2001 National Institute of Child Health and Human Development (NICHD) Diagnostic Criteria, 2016 Revisions of NICHD Criteria, and 2019 Neonatal Research Network Jensen Grading).

Results

A total of 451 infants (29.3%) had BPD (BPD group) while 1088 (70.7%) did not (non-BPD group), and the overall incidence of BPD was 29.3%. The most relevant risk factors associated with a higher risk of developing BPD identified in the multivariate logistic regression analysis were appropriate weight for gestational age (adjusted OR (aOR) 3.67, 95%CI 2.02-6.67, P < 0.001), presence of patent ductus arteriosus (PDA) (aOR 2.61, 95%CI 1.86-3.66, P < 0.001), late-onset sepsis (aOR 2.16; 95%CI 1.29-3.62; P = 0.003), and use of invasive ventilation (aOR 1.90; 95%CI 1.35-2.69; P < 0.001). The most relevant independent risk factors associated with a higher risk for developing moderate-severe BDP were postnatal steroids (aOR 7.12, 95%CI 3.77-13.44, P < 0.001), use of inhaled nitric oxide (aOR 3.65, 95%CI 1.48-9.01, P = 0.005), use of invasive ventilation (aOR 2.13, 95%CI 1.13-4.00, P = 0.019), late-onset sepsis (aOR 2.07, 95%CI 1.10-3.91, P = 0.025), and male sex (aOR 2.04, 95%CI 1.24-3.36, P = 0.005). The difference in the distribution of BPD severity across the three different definitions of NICHD was significant (P < 0.001).

Conclusion

The results of this study showed that the incidence of BPD remained high in infants born at ≤ 32 weeks of gestational age and birth weight <1500 g with appropriate weight for gestational age. The presence of PDA at birth or first echocardiography, late-onset sepsis, and use of invasive ventilation were significant risk factors for the incidence of BPD. The identification of risk factors will contribute to the implementation of lung-protective strategies for at-risk infants who may benefit from potential preventive therapy.

## Introduction

Bronchopulmonary dysplasia (BPD) is a chronic lung disease that primarily affects premature infants following respiratory support for acute respiratory distress at birth [1]. This condition has been widely recognized as a heterogeneous syndrome that results from a complex interaction between genetic, prenatal, and postnatal factors and leads to inflammation, variable lung injury, and repair with developmental arrest in immature lungs [1,2].

Despite marked advances in neonatal care over the past several decades such as antenatal steroids, surfactant therapy, and new ventilator strategies, BPD remains the most common prematurity-related complication [1,3]. While few studies have demonstrated an annual decline in the incidence of BPD [4], the majority have reported that the incidence of BPD has not declined or has, in fact, increased due to increased survival of very low gestational age (GA) infants [5]. On the other hand, BPD pathophysiology has shifted in parallel to changes in clinical practice over the past decades [5,6]. The diffuse fibroproliferative lung disease that first characterized the classic BPD in the pre-surfactant era (old BPD phenotype) has become a prominent growth arrest of the pulmonary vasculature and alveolar simpliﬁcation in the post-surfactant era, or "new BPD" [2,5]. This new BPD phenotype occurs most commonly in infants born before 28 weeks of gestation and whose birth weight is less than 1000 g [2,6].

BPD has a treatment-based definition [7]; and thus, along with advances in treatment modalities and accompanying changes in BPD pathophysiology, several refinements in BPD definitions have evolved [8]. The most popular definition is the National Institute of Child Health and Human Development (NICHD) consensus definition from 2001 [9] (revised in 2016 [10]); since then, a recent 2019 definition based on Neonatal Research Network (NRN) data by Jensen et al. has been introduced [11]. The use of various definitions of BPD across centers has had an impact on the incidence of BPD [12,13], with studies from multiple centers reporting that the incidence of BPD in infants < 28 weeks of GA fluctuated between 10% and 89% (Europe: 10-73%, North America: 18-89%, Asia: 18- 82%) [14]. Others have shown the incidence rate of BPD in premature infants < 32 weeks' gestation to vary between 12% and 32% [5].

The existing literature describes several risk factors associated with an increased incidence of BPD [3,15], with the strongest predictors being lower GA and birth weight [5]. Others include male sex, intrauterine growth restriction, sepsis, patent ductus arteriosus (PDA), necrotizing enterocolitis (NEC), pulmonary hypertension, and prolonged ventilator-induced injury [5,14,16,17]. However, the findings of these studies were inconsistent, reflecting the differences in the characteristics of the cohorts and definitions used. Early identification of the risk factors for BPD, particularly the moderate and severe forms, would probably permit the identification of at-risk patients who would likely benefit from postnatal management and possible future preventive therapy.

BPD predisposes survivors to significant pulmonary, cardiovascular, and neurodevelopmental morbidities and high mortality [1,18]. Children with this condition have a higher risk of chronic respiratory morbidity that manifests as asthma-like symptoms and lower lung function than term-born children [19,20]. Studies have reported an increased use of bronchodilators and a need for supplemental oxygen, and up to 50% of children with BPD are re-hospitalized during the first two years of life [20,21]. A meta-analysis reported a 20% prevalence of pulmonary hypertension among infants with BPD, and up to 40% among infants with severe BPD [22]. Other cardiovascular abnormalities associated with BPD have also been described, including left ventricular hypertrophy, pulmonary vein stenosis, and systemic-to-pulmonary collateral vessels [23]. Furthermore, affected infants are at greater risk of developing neurodevelopmental disorders including cerebral palsy and mental and psychomotor disabilities, and vision and hearing deficits are greater in infants with BPD than in those without BPD [19,24].

Therefore, this study aimed to determine the incidence of BPD among infants born at ≤ 32 weeks GA at our neonatal unit and to identify risk factors for the development of BPD and moderate-severe BPD.

## Materials and methods

We conducted a retrospective observational cohort study by reviewing the data from a neonatal database (Vermont Oxford Network). This database contains information on all infants admitted to the neonatal intensive care unit (NICU) at the Women’s Wellness and Research Center (WWRC), Hamad Medical Corporation (HMC), Doha, Qatar. The NICU in the WWRC is the main specialized center for women and newborn health services in Qatar.

The study was approved by the Institutional Review Board of Hamad Medical Corporation, Doha, Qatar (approval number: MRC-01-21-177). The requirement for signed informed consent was waived owing to the study’s retrospective observational nature. All methods were performed in accordance with the Declaration of Helsinki. All patient data were anonymous, and personal identifiers were excluded from data collection forms.

Inclusion and exclusion criteria

Infants born at ≤ 32 weeks of gestation with birth weights of ≤ 1500 grams in this hospital and admitted to the NICU between January 1, 2017, and December 31, 2020, were included in the study. We excluded infants with a diagnosis of congenital complex heart disease, congenital diaphragmatic hernia, congenital pulmonary anomalies, congenital infections, genetic disorders, chromosomal anomalies, and those who died before 28 days of life. However, infants with a specific diagnosis of PDA or patent foramen ovale were not excluded, as these conditions are highly prevalent among preterm infants who have structurally normal hearts.

Data collection

Relevant maternal, perinatal, and neonatal data were collected from the neonatal database to characterize the demographic and clinical characteristics as well as respiratory support. Baseline demographic characteristics of the enrolled preterm infants collected included GA, sex, birth weight, weight for GA, mode of delivery (cesarean or vaginal), multiple births, maternal diabetes, maternal pre-eclampsia, suspected chorioamnionitis, antenatal steroids, intubation and surfactant during resuscitation, Apgar score at one and five minutes, PDA, pulmonary hypertension by echocardiography at 36 weeks GA or prior to discharge, intraventricular hemorrhage (IVH), necrotizing enterocolitis (NEC), retinopathy of prematurity (ROP), sepsis, inhaled nitric oxide, and type of respiratory support. The length of stay in the NICU, BPD occurrences, BPD classifications, and deaths were also recorded. Univariate and multivariate logistic regression analyses were performed to identify potential factors and predictors and their possible associations with the development of BPD and moderate-severe BPD.

Outcomes

The primary outcomes of this study were to determine the incidence of BPD among premature infants born at ≤ 32 weeks GA and the proportion of moderate-severe BPD. The secondary outcome was to estimate the potential risk factors that may contribute to the development of BPD and moderate-severe BPD.

Definitions

GA was determined from the medical charts and calculated based on the last menstrual cycle of the mother during prenatal follow-up. Surfactant during resuscitation was defined as surfactant administered during the first hour of life (golden hour). Sepsis was defined as the presence of a single positive blood culture and/or clinical signs of infection. Early-onset sepsis was defined as sepsis occurring before 72 hours of life. Late-onset sepsis was defined as sepsis occurring after 72 hours of life.

Supplemental oxygen was defined as oxygen administered either via a nasal cannula or added to invasive or non-invasive ventilation; oxygen requirements were assessed at 36 weeks GA. Invasive respiratory support included high-frequency oscillatory and conventional mechanical ventilation. Non-invasive respiratory support included bilevel positive airway pressure, continuous positive airway pressure (CPAP), and a high-flow nasal cannula used as the maximum mode of respiratory support required during the NICU stay.

BPD was defined based on The National Institute of Child Health and Human Development (NICHD) consensus definition in 2001 as follows: no BPD if not receiving O2 for 28 days; mild BPD if receiving oxygen (O2) support for ≥ 28 days but not at 36 weeks postconceptional age; moderate BPD if receiving O2 for ≥ 28 days plus treatment with < fraction of inspired oxygen (FiO2) 30% at 36 weeks postconceptional age, and severe BPD if receiving O2 for ≥ 28 days plus treatment with ≥ FiO2 30% or positive pressure at 36 weeks postconceptional age [9]. The 2016 Revisions of the NICHD criteria were updated to include the need for radiographic evidence of pulmonary parenchymal disease. BPD severity was reclassified into grades I, II, and III according to respiratory support and FiO2 at 36 weeks GA. The revision added a new category, IIIA, for early death (between 14 days postnatal age and 36 weeks) due to persistent parenchymal lung disease and respiratory failure that cannot be attributed to other neonatal morbidities [10]. The 2019 BPD definition is based on neonatal research network (NRN) data by Jensen et al., and is only dependent on the mode of respiratory support and not the degree of oxygen supplementation at 36 weeks GA [11]. Grade I was defined as the use of a nasal cannula at a flow rate < 2 L/min; Grade II as nasal CPAP, non-invasive intermittent PPV, or a nasal cannula with a flow rate ≥ 2 L/min; and Grade III as invasive intermittent PPV.

Statistical analysis

Descriptive statistics were used to summarize and determine the sample characteristics and distribution of participants’ data. Normally distributed data and results were reported as means and SD, whereas skewed or non-normal data were reported as medians and ranges. Categorical data were summarized using frequencies and proportions. Associations between two or more qualitative data variables were assessed using the chi-square (χ2) test or Fisher’s exact test as appropriate. Quantitative data between two independent groups were analyzed using unpaired t-tests or Mann-Whitney U tests, as appropriate. Quantitative data and outcomes between two groups (BPD and non-BPD) and more than two independent groups (mild and moderate-severe) were analyzed using unpaired t-tests and one-way analysis of variance (ANOVA). Non-parametric Mann-Whitney U and Kruskal-Wallis tests were used for skewed or non-normally distributed data. The annual incidence trend was estimated, and statistical comparisons were performed using the extended Mantel-Haenszel chi-square test for linear trend analysis.

Univariate and multivariate logistic regression analyses were applied to determine and assess the potential factors and predictors associated with BPD and adjusted for potential factors. For the multivariate logistic regression models, predictor variables were included considering both statistical and clinical significance. The results of the logistic regression analysis are presented as odds ratios (ORs) with corresponding 95% confidence intervals (CIs). A receiver operating characteristic (ROC) curve was used to evaluate the discriminative ability (predictive accuracy of the developed logistic regression model) of potentially significant variables associated with the development of BPD or increasing its severity. Forest plots were constructed to depict the univariate and multivariate regression analyses of potential risk factors associated with moderate-severe BPD. All statistical P-values presented were two-tailed, and P-values <0.05 were considered statistically significant. All statistical analyses were performed using the IBM SPSS Statistics for Windows, Version 27.0 (Released 2020; IBM Corp., Armonk, New York, United States) and Epi Info software (Centers for Disease Control and Prevention, Atlanta, Georgia, United States).

## Results

**Figure 1:**
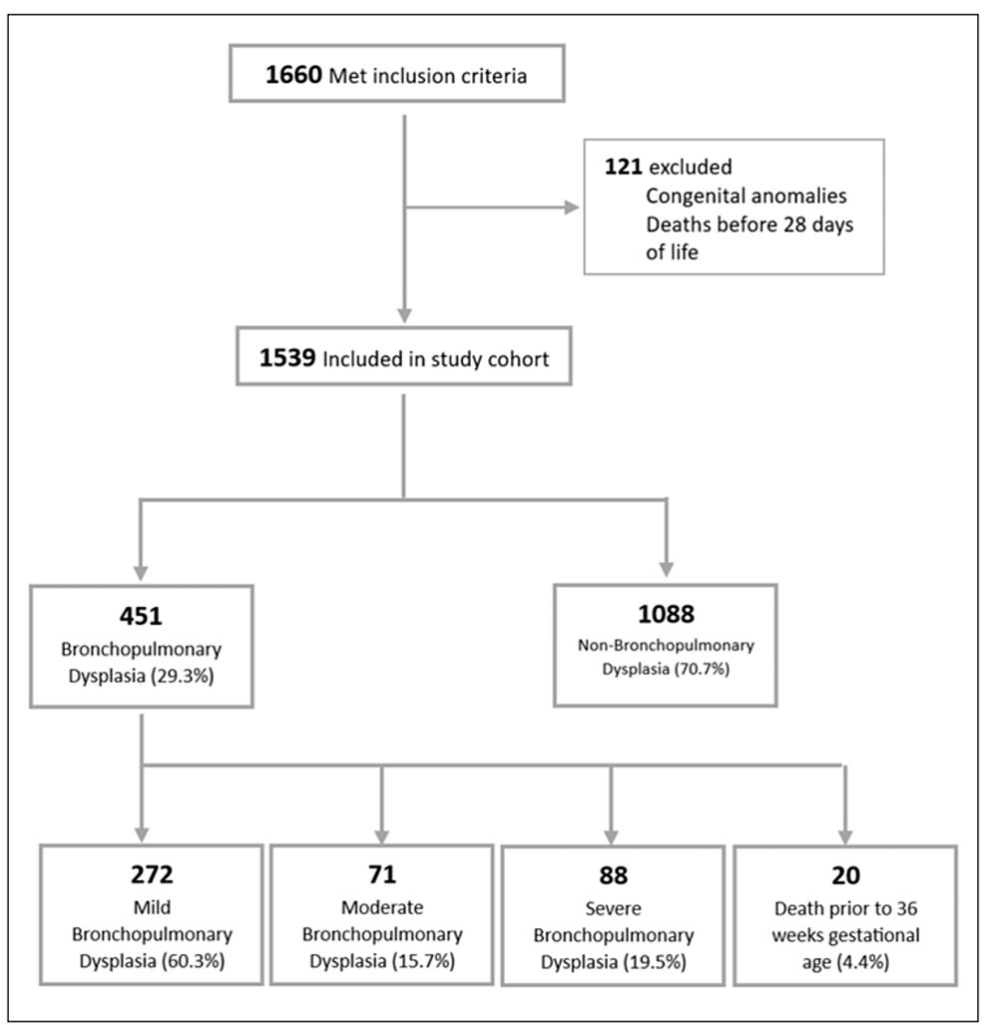
Study cohort population flow diagram and bronchopulmonary dysplasia severity distribution

During the study period, the Vermont-Oxford database included 1660 neonates born preterm at ≤ 32 weeks of gestation with a birth weight of ≤ 1500 g who were admitted to our NICU between January 2017 and December 2020. After applying the inclusion and exclusion criteria, 1539 eligible preterm infants were included in the BPD and non-BPD analyses (695 females, 45.2%). A total of 451 patients (29.3%) were diagnosed with BPD according to the 2001 NICHD Diagnostic Criteria, while 1088 (70.7%) did not meet the BPD definition (non-BPD group) (Figure 1).

Table 1 shows the patient demographics including antenatal and perinatal characteristics, encountered complications, and respiratory support data for the BPD and non-BPD groups.

**Table 1:** Patient demographics (BPD and non-BPD groups) BPD: bronchopulmonary dysplasia; SD: standard deviation; GA: gestational age; AGA: appropriate weight for gestational age; PDA: patent ductus arteriosus; NICU: neonatal intensive care unit * Reference group: Small for gestational age; ** Reference group: Non-Invasive respiratory support; ᶷ Respiratory support used as maximum support during NICU stay. All percentages (%) were computed using non-missing data values.

Variable	BPD Group (N=451)	Non-BPD Group (n=1088)	P-value
Antenatal Data			
Male gender, n (%)	245 (54.3%)	598 (55%)	0.819
Gestational age (weeks), Mean ± SD	26.38 ± 1.93	29 ± 2	<0.001
Birth weight (grams), Mean ± SD	935 ± 255	1371 ± 379	<0.001
AGA*, n (%)	424 (94%)	961 (88.3%)	0.001
Multiple birth, n (%)	152 (33.7%)	395 (36.3%)	0.332
Maternal diabetes, n (%)	92 (20.4%)	209/1086 (19.2%)	0.604
Maternal pre-eclampsia, n (%)	78 (17.3%)	193/1086 (17.7%)	0.823
Chorioamnionitis, n (%)	47 (10.4%)	63 (5.8%)	0.002
Antenatal steroids, n (%)	382 (84.7%)	858 (78.9%)	0.008
Perinatal Data			
Vaginal delivery, n (%)	161 (35.7%)	272 (25%)	<0.001
Apgar at 1 minute, Mean ± SD	5 ± 2	7 ± 2	<0.001
Apgar at 5 minutes, Mean ± SD	8 ± 2	9 ± 1	<0.001
Intubated during resuscitation, n (%)	304 (67.4%)	242 (22.2%)	<0.001
Surfactant during resuscitation, n (%)	220 (48.8%)	161 (14.8%)	<0.001
Hospital Course/Complications			
PDA at birth/first echocardiography, n (%)	254 (56.3%)	120/1084 (11%)	<0.001
Intraventricular hemorrhage (IVH)			<0.001
None - n (%)	325/450 (72.1%)	984 (90.4%)	
Grade I – IVH, n (%)	50/450 (11.1%)	64 (5.9%)	
Grade II – IVH, n (%)	49/450 (10.9%)	22(2%)	
Grade III – IVH, n (%)	11/450 (2.4%)	4 (0.4%)	
Grade IV – IVH, n (%)	15/450 (3.3%)	14 (1.3 %)	
Necrotizing enterocolitis (NEC), n (%)	47 (10.4%)	69 (6.3%)	0.297
Retinopathy of Prematurity (ROP) Stage			<0.001
None, n (%)	279 (61.9%)	1012 (93%)	
Stage I, n (%)	46 (10.2%)	54 (5%)	
Stage II, n (%)	76 (16.9%)	18 (1.7%)	
Stage III, n (%)	50 (11.1%)	4 (0.4%)	
Sepsis			<0.001
None, n (%)	368 (81.6%)	1033 (94.9%)	
Early onset sepsis, n (%)	6 (1.3%)	14 (1.3%)	
Late onset sepsis, n (%)	77 (17.1%)	41 (3.8%)	
Respiratory Support Data			
Inhaled nitric oxide, n (%)	50 (11.1%)	22 (2%)	<0.001
Invasive ventilation ** ᶷ, n (%)	317 (70.3%)	247 (22.7%)	<0.001
Duration of mechanical ventilation (Days), Median (Range)	14 (2-200)	3 (2 - 32)	<0.001
Length of NICU stay (Days), Mean ± SD	86 ± 43	32 ± 19	<0.001

The overall incidence of BPD among preterm infants (≤32 weeks GA) at our institute was 29.3% (451/1539) (95%CI 27.1%-31.6%). Across the four years of the study period, the incidence of BPD was 28.7% (118/410), 23.3% (95/407), 33.3% (116/348), and 32.6% (122/374) in 2017, 2018, 2019, and 2020, respectively. The annual difference in the incidence estimates across the included years was statistically significant according to the extended Mantel-Haenszel chi-square test for linear trend analysis (c2 linear trend = 4.06, P = 0.044).

The mean GA (29 ± 2 weeks versus 26.38 ± 1.93 weeks) and birthweight (1371 ± 379 grams versus 935 ± 255 grams) were significantly higher in the non-BPD group than in the BPD group (P < 0.001).

Among the infants with BPD, the rates of intubation and surfactant administration in the delivery room were also significantly higher than those in the non-BPD group (P < 0.001); in total, 67.4% of the neonates with BPD versus 22.2% of the neonates without BPD required intubation in the delivery room. Additionally, 48.8% of the neonates with BPD versus 14.8% of the neonates without it required surfactants during resuscitation in the first hour of life.

The rates of appropriate weight for GA (AGA) status, chorioamnionitis, antenatal steroid use, vaginal delivery, use of inhaled nitric oxide, and use of invasive respiratory support were significantly higher in the BPD group (P < 0.05). However, male sex, multiple births, maternal diabetes, maternal preeclampsia, and NEC did not differ significantly between the BPD and non-BPD groups. The rates of other prematurity comorbidities (intraventricular hemorrhage, retinopathy of prematurity, patent ductus arteriosus, and sepsis) were significantly higher in infants with BPD than in those without BPD (P <0.001).

Only 235 (52.1%) premature infants with BPD were assessed using echocardiography for pulmonary hypertension at 36 weeks GA or prior to discharge at the discretion of the primary physician. Of these, 33 (7.3%) had pulmonary hypertension. However, none of the babies in the non-BPD group underwent echocardiography prior to discharge or at 36 weeks GA. Death during NICU stay was observed in 26 (5.8%) infants in the BPD group and 74 (6.8%) in the non-BPD group. The difference between the two groups was not statistically significant (P = 0.452).

Univariate analysis performed to evaluate potential predictors and risk factors associated with BPD revealed that AGA (unadjusted OR 2.07, 95%CI 1.34-3.19, P = 0.001), intubation (unadjusted OR 7.23, 95%CI 5.66-9.22, P < 0.001), surfactant administration (unadjusted OR 5.48, 95%CI 4.27-7.03, P < 0.001) during resuscitation, use of inhaled nitric oxide (unadjusted OR 6.04; 95%CI 3.61-10.11; P < 0.001), and invasive ventilation (unadjusted OR 8.05, 95%CI 6.29-10.31, P < 0.001) were significantly associated with a higher risk of developing BPD. Conversely, higher GA (unadjusted OR 0.53, 95%CI 0.50-0.57, P < 0.001), and birth weight (unadjusted OR 0.99, 95%CI 0.99-0.99, P < 0.001) were associated with a lower risk of developing BPD.

The most relevant risk factors associated with a higher risk of developing BPD identified in the multivariate logistic regression analysis were AGA status (adjusted OR (aOR) 3.67, 95% CI 2.02 - 6.67, P < 0.001), presence of PDA at birth or first echocardiography (aOR 2.61, 95% CI 1.86 - 3.66, P < 0.001), late-onset sepsis (aOR 2.16, 95%CI 1.29-3.62, P = 0.003), and use of invasive ventilation (aOR 1.90, 95%CI 1.35-2.69, P < 0.001). Vaginal delivery (aOR 0.67, 95%CI 0.46-0.97, P = 0.034) and higher GA (aOR 0.76; 95%CI 0.68-0.85; P < 0.001) were less likely to be associated with BPD. Table 2 summarizes the results of the univariate and multivariate logistic regression analyses that determined the potential predictors and risk factors associated with the development of BPD.

**Table 2:** Logistic regression analysis with potential factors and predictors associated with BPD BPD: bronchopulmonary dysplasia; OR: odds ratio, 95%CI: 95% confidence interval for odds ratio; SD: standard deviation; GA: gestational age; AGA: appropriate weight for gestational age; PDA: patent ductus arteriosus ¥ Percentages were calculated against the total number of each variable; * Reference group: Small for gestational age; ** Reference group: Non-invasive respiratory support All percentages (%) were computed using non-missing data values.

Variable		Univariate analysis		Multivariate analysis	
	BPD, n (%) ¥	Unadjusted OR (95% Cl)	P value	Adjusted OR (95% CI)	P value
Antenatal Data					
Male Gender, n (%)	245 (29.1%)	0.97 (0.78 - 1.21)	0.819	1.03 (0.77 - 1.39)	0.828
Gestational age (weeks), mean ± SD	26.38 ± 1.93	0.53 (0.50 - 0.57)	<0.001	0.76 (0.68 - 0.85)	<0.001
Birth weight (grams), mean ± SD	935 ± 255	0.99 (0.99 – 0.99)	<0.001	0.99 (0.99 - 0.99)	<0.001
AGA*, n (%)	424 (30.6%)	2.07 (1.34 - 3.19)	0.001	3.67 (2.02 - 6.67)	<0.001
Multiple Birth, n (%)	152 (27.8%)	0.89 (0.70 - 1.12)	0.332	0.78 (0.56 - 1.08)	0.132
Maternal Diabetes, n (%)	92 (30.6%)	1.07 (0.81 - 1.41)	0.604	1.29 (0.90 - 1.87)	0.169
Maternal Pre-eclampsia, n (%)	78 (28.8%)	0.96 (0.72 - 1.29)	0.823	0.86 (0.57 - 1.31)	0.489
Chorioamnionitis, n (%)	47 (42.7%)	1.89 (1.28 - 2.81)	0.002	0.93 (0.54 - 1.61)	0.804
Antenatal steroids, n (%)	382 (30.8%)	1.48 (1.11 - 1.99)	0.009	1.55 (1.04 - 2.31)	0.033
Perinatal Data					
Vaginal delivery, n (%)	161 (37.2%)	1.66 (1.31 -2.11)	<0.001	0.67 (0.46 - 0.97)	0.034
Apgar at 1 minute, mean ± SD	5 ± 2	0.72 (0.69 - 0.76)	<0.001	0.97 (0.88 - 1.08)	0.622
Apgar at 5 minutes, mean ± SD	8 ± 2	0.62 (0.56 - 0.67)	<0.001	1.06 (0.90 - 1.24)	0.501
Intubated during resuscitation, n (%)	304 (55.7%)	7.23 (5.66 - 9.22)	<0.001	1.29 (0.84 - 1.96)	0.245
Surfactant during resuscitation, n (%)	220 (57.7%)	5.48 (4.27 - 7.03)	<0.001	1.51 (1.04 - 2.20)	0.032
Hospital Course/Complications					
PDA at birth/first echocardiography, n (%)	254 (67.9%)	10.36 (7.94 – 13.51)	<0.001	2.61 (1.86 - 3.66)	<0.001
Necrotizing Enterocolitis (NEC), n (%)	47 (40.5%)	1.72 (1.17 – 2.53)	0.006	0.53 (0.32 - 0.90)	0.019
Sepsis			<0.001		0.013
None, n (%)	368 (26.3%)	1 (Reference)		1 (Reference)	
Early onset sepsis, n (%)	6 (30%)	1.20 (0.46 - 3.15)	0.707	0.92 (0.28 - 3.01)	0.885
Late onset sepsis, n (%)	77 (65.3%)	5.27 (3.54 - 7.84)	<0.001	2.16 (1.29 - 3.62)	0.003
Respiratory support data					
Inhaled Nitric Oxide, n (%)	50 (69.4%)	6.04 (3.61 – 10.11)	<0.001	1.01 (0.52 - 1.94)	0.998
Invasive Ventilation **, n (%)	317 (56.2%)	8.05 (6.29 - 10.31)	<0.001	1.90 (1.35 - 2.69)	<0.001

The NICHD 2001 definition was used to define the BPD group. Among infants with BPD, 60.3%, 15.7%, and 19.5% had mild, moderate, and severe BPD, respectively. Twenty neonates (4.4%) were defined as having BPD but were not classified because they required oxygen support for 28 days of life but passed away prior to the assessment point at 36 weeks GA. Therefore, these 20 neonates were excluded from the severity analysis.

The rates of intubation and surfactant administration during resuscitation, postnatal steroid administration, inhaled nitric oxide, and use of supplemental oxygen at 36 weeks GA were significantly higher in the severe BPD group. Prematurity-related comorbidities such as PDA, pulmonary hypertension, and ROP were also significantly higher in the severe BPD group. Additionally, the duration of mechanical ventilation and NICU stay were significantly longer in the severe BPD group (P < 0.001). Conversely, the GA, birth weight, multiple births, and Apgar scores were significantly lower in the severe BPD group. Thirty-three infants had pulmonary hypertension prior to discharge or at 36 weeks GA. Across the severity classifications, 22/88 infants with severe BPD, 2/71 with moderate BPD, 7/272 with mild BPD, and 2/20 with unclassified BPD had evidence of pulmonary hypertension on echocardiography.

Patient demographics across BPD severity classifications according to the NICHD 2001 definition are summarized in Table 3.

**Table 3:** Demographics across BPD severity classifications according to NICHD 2001 definition. 20 patients were excluded from this analysis and were not classified due to death prior to 36 weeks gestational age. NICHD: National Institute of Child Health and Human Development; BPD: bronchopulmonary dysplasia; SD: standard deviation; GA: gestational age; AGA: appropriate weight for gestational age; PDA: patent ductus arteriosus; NICU: neonatal intensive care unit ᶺ Evident by echocardiography at 36 weeks GA or prior to discharge; * Reference group: Small for gestational age; ** Reference group: Non-Invasive respiratory support; ᶷ Respiratory support used as maximum support during NICU stay All percentages (%) were computed using non-missing data values.

Variable	Mild BPD N=272	Moderate BPD N=71	Severe BPD N=88	P Value
Antenatal Data				
Male Gender, n (%)	139 (51.1%)	41 (57.7%)	57 (64.8%)	0.071
Gestational age (weeks), mean ± SD	27 ± 2	26 ± 2	26 ± 2	0.011
Birth weight (grams), mean ± SD	983 ± 249	898 ± 227	835 ± 256	<0.001
AGA*, n (%)	261 (96%)	65 (91.5%)	79 (89.8%)	0.069
Multiple Birth, n (%)	103 (37.9%)	21 (29.6%)	20 (22.7%)	0.025
Maternal Diabetes, n (%)	61 (22.4%)	15 (21.1%)	12 (13.6%)	0.200
Maternal Pre-eclampsia, n (%)	47 (17.3%)	13 (18.3%)	14 (15.9%)	0.920
Chorioamnionitis, n (%)	29 (10.7%)	6 (8.5%)	10 (11.4%)	0.820
Antenatal Steroids, n (%)	230 (84.6%)	61 (85.9%)	73 (83%)	0.870
Perinatal Data				
Vaginal delivery, n (%)	89 (32.7%)	28 (39.4%)	38 (43.2%)	0.165
Apgar at 1 minute, mean ± SD	6 ± 2	6 ± 2	5 ± 2	0.008
Apgar at 5 minutes, mean ± SD	8 ± 1	8 ± 1	7 ± 2	0.018
Intubated during resuscitation, n (%)	171 (62.9%)	48 (67.6%)	70 (79.5%)	0.015
Surfactant during resuscitation, n (%)	129 (47.4%)	26 (36.6%)	53 (60.2%)	0.011
Hospital Course/Complications				
PDA at birth/first echocardiography, n (%)	128 (47.1%)	42 (59.2%)	68 (77.3%)	<0.001
Pulmonary hypertension ᶺ, n (%)	7/114 (6.1%)	2/47 (4.3%)	22/70 (31.4%)	<0.001
Intraventricular Hemorrhage (IVH)				0.180
None, n (%)	208 (76.5%)	49 (69%)	54 (61.4%)	
Grade I – IVH, n (%)	27 (9.9%)	8 (11.3%)	13 (14.8%)	
Grade II – IVH, n (%)	28 (10.3%)	8 (11.3%)	12 (13.6%)	
Grade III – IVH, n (%)	4 (1.5%)	3 (4.2%)	4 (4.5%)	
Grade IV – IVH, n (%)	5 (1.8%)	3 (4.2%)	5 (5.7%)	
Necrotizing Enterocolitis (NEC), n (%)	19 (7%)	9 (12.7%)	13 (14.8%)	0.059
Retinopathy of prematurity (ROP) stage				<0.001
None, n (%)	188 (69.1%)	39 (54.9%)	36 (40.9%)	
Stage I, n (%)	28 (10.3%)	6 (8.5%)	11 (12.5%)	
Stage II, n (%)	44 (16.2%)	12 (16.9%)	18 (20.5%)	
Stage III, n (%)	12 (4.4%)	14 (19.7%)	23 (26.1%)	
Sepsis				0.012
None, n (%)	234 (86%)	56 (78.9%)	64 (72.7%)	
Early onset sepsis, n (%)	5 (1.8%)	1 (1.4%)	0 (0%)	
Late onset sepsis, n (%)	33 (12.2%)	14 (19.7%)	24 (27.3%)	
Respiratory support data				
Postnatal corticosteroids, n (%)	22 (8.1%)	17 (23.9%)	52 (59.1%)	<0.001
Inhaled Nitric Oxide, n (%)	10 (3.7%)	5 (7%)	26 (29.5%)	<0.001
Invasive ventilation ** ᶷ, n (%)	161 (59.2%)	51 (71.8%)	85 (96.6%)	0.124
Supplemental Oxygen at 36 weeks GA, n (%)	79 (28.1%)	55 (77.5%)	74 (84.1%)	<0.001
Duration of mechanical ventilation (Days), median (Range)	7 (2 – 66)	14 (2 – 165)	34 (2 – 200)	<0.001
Length of NICU stay (Days), mean ± SD	70.9 ± 24.8	98.8 ± 28.6	135.4 ± 56.6	<0.001

**Figure 2:**
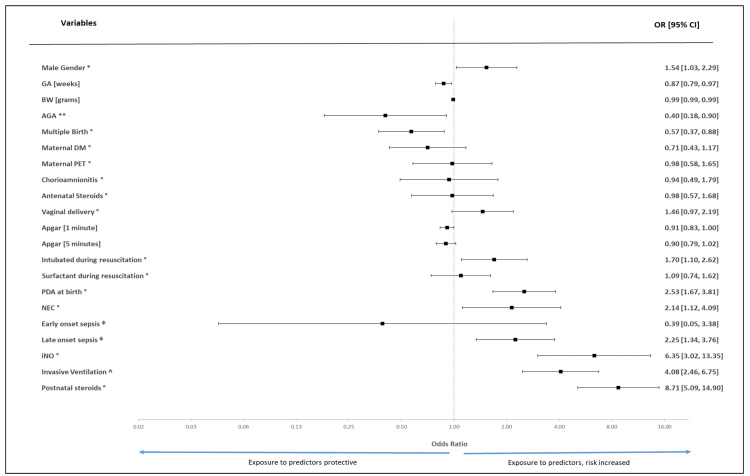
Forest plot to depict the effect size computed using univariate logistic regression (using dichotomous outcome variable: moderate-severe versus mild BPD) analysis determining potential predictors and risk factors in developing moderate-severe bronchopulmonary dysplasia (BPD). OR: odds ratio; 95% CI: 95% confidence interval for odds ratio; GA: gestational age; BW: birthweight; AGA: appropriate weight for gestational age; DM: diabetes mellitus; PET: preeclampsia toxemia; PDA: patent ductus arteriosus; iNO: inhaled nitric oxide * Reference group: Female gender; ** Reference group: Small for gestational age; ° Reference group: Absence of parameter; ᶲ Reference group: No sepsis; ^ Reference group: Non-invasive respiratory support

Univariate and multivariate logistic regression analyses were performed to evaluate potential factors and predictors along with their possible association with moderate-severe BPD. In the univariate analysis, male sex, intubation during resuscitation, presence of PDA at birth, NEC, late-onset sepsis, use of inhaled nitric oxide, use of postnatal steroids, and use of invasive ventilation were significantly associated with moderate-severe BPD. Low GA, infants with AGA status, multiple births, and Apgar score at 1 minute were associated with a lower risk of developing moderate-severe BPD. Figure 2 shows a forest plot of the univariate logistic regression analysis used to determine potential predictors and risk factors associated with moderate-severe BPD.

**Figure 3:**
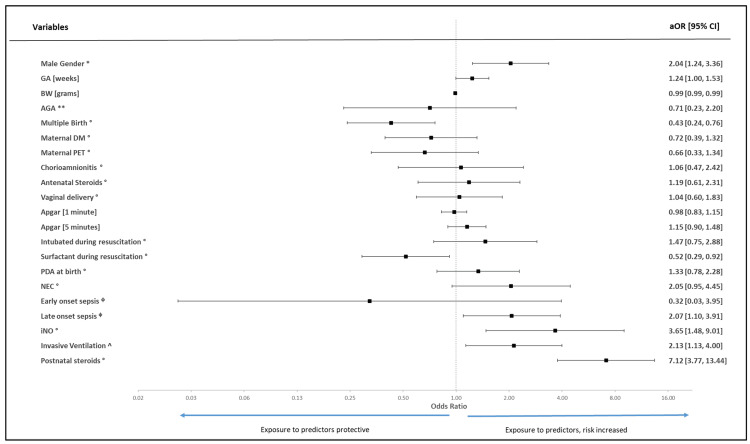
Forest plot to depict the effect size computed using multivariate logistic regression (using dichotomous outcome variable: moderate-severe versus mild BPD) analysis determining potential predictors and risk factors in developing moderate-severe bronchopulmonary dysplasia (BPD). aOR: adjusted odds ratio; 95%CI: 95% confidence interval for odds ratio; GA: gestational age; BW: birthweight; AGA: appropriate weight for gestational age; DM: diabetes mellitus; PET: preeclampsia toxemia; PDA: patent ductus arteriosus; iNO: inhaled nitric oxide * Reference group: Female gender; ** Reference group: Small for gestational age; ° Reference group: Absence of parameter; ᶲ Reference group: No sepsis

The most relevant independent risk factors associated with a higher risk for developing moderate-severe BDP identified in the multivariate logistic regression analysis were postnatal steroids (aOR 7.12, 95%CI 3.77-13.44, P < 0.001), use of inhaled nitric oxide (aOR 3.65, 95%CI 1.48-9.01, P = 0.005), use of invasive ventilation (aOR 2.13, 95%CI 1.13-4.00, P = 0.019), late-onset sepsis (aOR 2.07, 95%CI 1.10-3.91, P = 0.025), and male sex (aOR 2.04, 95%CI 1.24-3.36, P = 0.005). In contrast, multiple births (aOR 0.43, 95%CI 0.24-0.76, P = 0.004) and surfactant administration during resuscitation (aOR 0.52, 95%CI 0.29-0.92, P = 0.024) were associated with a lower risk of developing moderate-severe BPD. Figure 3 shows the forest plot of the multivariate logistic regression analysis for the risk factors for developing moderate-severe BPD compared to mild BPD.

The discriminative ability of potentially significant predictors (observed in multivariate analysis) in predicting BPD was evaluated by a computed predictive logistic regression model and found to be good with an area under the ROC curve value of 0.892 (95%CI 0.88-0.91), indicating an excellent fit for the regression model.

Similarly, the discriminative ability of the significant predictors (observed in multivariate analysis) in predicting BPD severity was also found to be good, with an area under the ROC curve (AUC) value of 0.803 (95%CI 0.76-0.85), again indicating that the regression model developed demonstrated a good fit.

**Figure 4:**
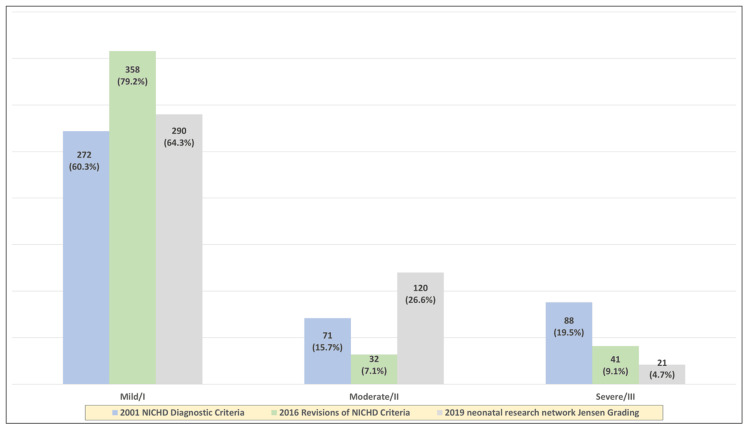
Distribution of bronchopulmonary dysplasia severity across the different definitions of National Institute of Child Health and Human Development (NICHD).

According to the 2016 revision of the NICHD criteria, 358 (79.2%), 32 (7.1%), 41 (9.1%), and 20 (4.4 %) infants were classified as having classes I, II, III, and IIIA/lethal BPD, respectively. According to the 2019 definition, 290 (64.3%), 120 (26.6%), and 21 (4.7%) patients were classified as classes I, II, and III, respectively. Twenty infants (4.4%) in both groups were classified as having lethal BPD/IIIA according to the 2016 definition but were considered unclassified according to the 2001 and 2019 definitions. The distribution of BPD severity across different NICHD definitions is shown in Figure 4. The differences in each of the BPD classes (mild/I, moderate/II, and severe/III) across the three definitions were significant (P < 0.001).

## Discussion

BPD remains the most common prematurity-related complication and is associated with substantial long-term multisystem morbidity and high mortality [20]. BPD was diagnosed in 29.3% of our cohort of premature infants born at ≤ 32 weeks of gestation. Furthermore, we found the incidence of BPD across the four-year study period to be 28.7%, 23.3%, 33.3%, and 32.6% in 2017, 2018, 2019, and 2020, respectively, and the difference was significant. This incidence is consistent with that reported in the literature; specifically, it is similar to the reported BPD rate of 33.1% among very low birth weight (VLBW) infants born between 22 and 29 weeks GA survivors reported by Wannasiri et al. in their large multicenter study [25]. The incidence in the current study is also similar to the regional incidence of BPD reported by Alshehri et al., who found the prevalence of BPD among Saudi preterm infants to be 30.5% [26]. However, Galuh et al. found a lower incidence of BPD in infants with birth weights < 1500 g (19%), with the highest fraction observed in the < 28 weeks of GA and < 1000 g group [14]. This observed variability primarily reflects the heterogeneity among cohorts, methodological differences, variations in clinical practices, and differences in BPD definition used between centers.

Extreme prematurity and extremely low birth weight remain the strongest predictors of BPD owing to the underdevelopment of lung structures [2,5]. In agreement with the previous studies that have demonstrated an inverse relationship between the incidence of BPD and GA and birthweight [14,27], our results showed a mean GA of 26.38 ± 1.93 weeks and a mean birthweight of 935 ± 255 grams in the BPD group, which were significantly lower than those in the non-BPD group. These values indicate that the incidence of BPD is directly correlated with a lower GA and birth weight.

In small-for-GA (SGA) infants, chronic stress exposure induces lung maturation, which reduces the severity of RDS and the risk of BPD. Our results showed that AGA increased the incidence of BPD with an aOR of 3.67 (95%CI 2.02-6.67, P < 0.001) compared with that found in SGA infants. This observation is similar to that reported by Martin et al., who showed an absolute risk of BPD that was higher in the AGA group than in the SGA group [14]. In contrast, other studies have suggested that SGA preterm neonates have a higher risk of BPD than AGA neonates [17]. It has been posited that biological derangements that result in SGA can impact lung growth and increase the risk of BPD among SGA infants [5]. The different characteristics of the enrolled preterm infants may partially explain this discrepancy; therefore, extensive multicenter studies with a high proportion of extremely low gestational-age infants are warranted to accurately elucidate the relationship between GA and BPD.

Previous studies have linked PDA with BPD as an essential risk factor [14]. Our results suggest that the presence of PDA at birth or first echocardiography increased the risk of BPD (aOR 2.61, 95%CI 1.86-3.66, P < 0.001). This finding is similar to that of another study that demonstrated that PDA increased the risk of BPD by 4.9 among infants with low birth weight and gestational weeks [28]. Additionally, the effect of volutraumatic injury associated with BPD on the developing lungs may contribute to BPD outcomes. However, the precise role of PDA in the incidence of BPD is debatable in the literature, as early closure of PDA, either surgically or with medication, does not decrease the development of BPD [6,27].

Antenatal corticosteroid administration has been proposed to reduce the severity of respiratory distress syndrome. Therefore, it reduces the risk of developing BPD in preterm infants [6]. However, previous studies have suggested that antenatal corticosteroid administration does not modify the risk of BPD due to the associated increased survival of extremely preterm infants exposed to antenatal steroids [5]. In one study, a significantly higher proportion of the BPD group received antenatal corticosteroids than those in the non-BPD group (14.4% vs 3.3%, P < 0.01). However, this was not a significant risk factor for BPD [28]. In contrast, in our study, antenatal corticosteroid administration was higher in the BPD group than in the non-BPD group, but it was identified as a risk factor for BPD (aOR 1.55, 95%CI 1.04-2.31, P = 0.033). Different cohort characteristics may have contributed to these contradictory findings.

Our study also found that intubation and surfactant administration during resuscitation were significant risk factors for BPD in univariate regression analysis. The close association between surfactant administration and RDS severity, which is a significant risk factor for BPD, may have contributed to this observation. However, previous studies have demonstrated a significant protective effect of postnatal surfactants against BPD [28].

While survival of extremely premature infants necessitates the use of mechanical ventilation, mechanical ventilation-induced barotrauma, biotrauma, and volutrauma lead to structural and inflammatory changes in the developing lungs. This technique significantly increases the risk of developing BPD in extremely low GA (ELGA) infants [5,27]. Martin et al. found that mechanical ventilation during the first two days of life was the most potent risk factor for BPD (OR = 7.74, P = 0.008) [14]. In our study, a higher proportion of patients in the BPD group (70.3%) received mechanical ventilation during their stay than in the non-BPD group (8.2 %), and the use of invasive ventilation increased the risk of BPD (aOR 1.90, 95%CI 1.35-2.69, P < 0.001). Another result of this study was that the protective effects of non-invasive respiratory support were demonstrated in our cohort, in agreement with previous studies in which CPAP decreased the risk of BPD [18,15]. Additionally, sepsis was taken into account in this study as it induces inflammation and lung injury and disrupts lung growth in vulnerable immature lungs [6,15]. Our results showed late-onset sepsis to be a risk factor for BPD (aOR 2.16, 95%CI 1.29-3.62, P = 0.003); however, no significant correlation was observed between early sepsis and BPD. These findings are consistent with those of studies that have established a link between late-onset sepsis and the risk of BPD but not with early sepsis [28]. In contrast, other studies have shown that early-onset sepsis predicted BPD [14]; however, these inconsistencies may be related to differences in the definitions of sepsis used in these studies.

Among the BPD groups in our study, 60.3% were classified as mild BPD, 15.7% as moderate BPD, and 19.5% as severe BPD according to the NICHD 2001 severity classification. In contrast, Yang et al. showed that of 250 preterm infants ≤ 32 weeks of GA with a diagnosis of mild, moderate, and severe BPD were 39 (15.6%), 185 (74.0%), and 26 (10.4%), respectively [17]. Another study of 266 ELGAs born at GA ≤ 28 weeks had moderate-severe BPD in 67% (145/216) of survivors at 36 weeks' gestation [27].

In our study, the independent risk factors that increased the incidence of moderate-severe BPD were male sex, invasive ventilation, inhaled nitric oxide, late-onset sepsis, and postnatal steroid use. In their univariate analysis, Geetha et al. demonstrated similar risk factors for moderate and severe BPD (sepsis, hemodynamically significant PDA, and mechanical ventilation) in addition to birthweight, air leak, and FiO2 requirement > 25%; however, day seven mechanical ventilation was the only significant independent risk factor for moderate and severe BPD in their study [27]. In contrast, our study demonstrated that multiple births and surfactant administration during resuscitation had protective effects against the development of moderate-severe BPD.

In another study, 12.1% of infants ≤32 weeks GA who developed BPD also developed late pulmonary hypertension (> 28 days after birth), which was associated with significantly increased mortality (36.4%) compared to those without late pulmonary hypertension (1.9%) [29]. In our cohort, only 235 (52.1%) premature infants with BPD were assessed for pulmonary hypertension at 36 weeks GA or before discharge. Of these, 33 (7.3%) had pulmonary hypertension, and among infants with moderate and severe BPD, 42 of 159 were not screened for pulmonary hypertension. Therefore, we emphasize screening for pulmonary hypertension among infants with moderate and severe BPD at our institute according to the recommendations of the Pediatric Pulmonary Hypertension Network (PPHNet) [30].

As reflected by the existing literature, the incidence of BPD varies according to the definition used. In their study, Gomez et al. compared Shennan’s definition with the consensus definition of BPD from the National Institutes of Health (NIH) workshop, and estimated the difference in BPD prevalence between the two criteria to be 32% (P < 0.0001). According to the NIH criteria, the prevalence of BPD is 80% higher and is a better predictor of oxygen requirement at discharge [13]. The variation in BPD severity classification was highlighted in our study; in our comparison between the 2016 Revisions of NICHD Criteria and the 2019 NRN Jensen Grading with the 2001 NICHD Diagnostic Criteria, the differences in each of the BPD classes (mild/I, moderate/II, and severe/III) across the three definitions were significant (P < 0.001). These findings emphasize the importance of standardizing the tools used to identify and classify BPD to standardize the incidence and classification of BPD.

Limitations

The most important limitations of our study are the relatively small sample size and the difficulty in eliminating confounding factors. Statistical significance was found between BPD and non-BPD groups. However, even with this sample size, the two groups had differences in several variables. Additionally, our study has a retrospective design which does not allow the confirmation of the causality of the associations. Longitudinal cohort studies are warranted to determine the accurate correlation between classification and long-term outcomes, which would allow the provision of ambulatory care for high-risk infants with BPD at the time of release.

## Conclusions

Our study showed that AGA, presence of PDA at birth or first echocardiography, late-onset sepsis, and use of invasive ventilation were significant risk factors for the incidence of BPD in infants with GA ≤ 32 weeks and birth weight < 1500 g. Furthermore, the incidence of BPD and its severity classification varies according to the definition used. Hence, standardizing tools to identify BPD is of paramount importance to accurately delineate its incidence and plan the multidisciplinary care required for high-risk infants with BPD.
